# Inferring Carbon Sources from Gene Expression Profiles Using Metabolic Flux Models

**DOI:** 10.1371/journal.pone.0036947

**Published:** 2012-05-14

**Authors:** Aaron Brandes, Desmond S. Lun, Kuhn Ip, Jeremy Zucker, Caroline Colijn, Brian Weiner, James E. Galagan

**Affiliations:** 1 Broad Institute of MIT and Harvard, Cambridge, Massachusetts, United States of America; 2 Department of Computer Science and Center for Computational and Integrative Biology, Rutgers University, Camden, New Jersey, United States of America; 3 Phenomics and Bioinformatics Research Centre, School of Mathematics and Statistics, and Australian Centre for Plant Functional Genomics, University of South Australia, Mawson Lakes, South Australia, Australia; 4 Department of Genetics, Harvard Medical School, Boston, Massachusetts, United States of America; 5 Department of Engineering Mathematics, University of Bristol, Bristol, United Kingdom; 6 Department of Biomedical Engineering and Department of Microbiology, Boston University, Boston, Massachusetts, United States of America; Tata Institute of Fundamental Research, India

## Abstract

**Background:**

Bacteria have evolved the ability to efficiently and resourcefully adapt to changing environments. A key means by which they optimize their use of available nutrients is through adjustments in gene expression with consequent changes in enzyme activity. We report a new method for drawing environmental inferences from gene expression data. Our method prioritizes a list of candidate carbon sources for their compatibility with a gene expression profile using the framework of flux balance analysis to model the organism’s metabolic network**.**

**Principal Findings:**

For each of six gene expression profiles for *Escherichia coli* grown under differing nutrient conditions, we applied our method to prioritize a set of eighteen different candidate carbon sources. Our method ranked the correct carbon source as one of the top three candidates for five of the six expression sets when used with a genome-scale model. The correct candidate ranked fifth in the remaining case. Additional analyses show that these rankings are robust with respect to biological and measurement variation, and depend on specific gene expression, rather than general expression level. The gene expression profiles are highly adaptive: simulated production of biomass averaged 94.84% of maximum when the *in silico* carbon source matched the *in vitro* source of the expression profile, and 65.97% when it did not.

**Conclusions:**

Inferences about a microorganism’s nutrient environment can be made by integrating gene expression data into a metabolic framework. This work demonstrates that reaction flux limits for a model can be computed which are realistic in the sense that they affect *in silico* growth in a manner analogous to that in which a microorganism’s alteration of gene expression is adaptive to its nutrient environment.

## Introduction

Our goal in this research is to draw inferences about an organism’s nutrient use from its pattern of gene expression. The key to the feasibility of our approach is the way microorganisms such as *Escherichia coli* make adaptations to optimize their growth when adequate nutrients are available. They can control the uptake and efflux of many metabolites through the expression of membrane-bound protein complexes called transporters. Since the biochemical reactions required to sustain life are largely catalyzed by enzymes, adjustment of the quantity and activity of enzymes provides another mode of control for microorganisms. An organism can therefore adjust to the presence of a desired nutrient by producing adequate transporters for its uptake and insuring that important internal biochemical reactions are not limited by insufficient enzyme activity.

In addition, the organism may restrict the expression of transporters and enzymes that favor the use of less preferred carbon sources. For example *E. coli* growing on glucose will, by use of the *lac* operon, severely restrict production of both the permease that would allow the transport of lactose into the organism and the enzyme beta-galactosidase required to catabolize lactose into glucose. Thus, in the presence of both glucose and lactose, the bacterium will consume all the available glucose before metabolizing the lactose [1].

The main contribution of this research is a method for prioritizing candidate nutrient conditions from gene expression data**.** We draw environmental inferences based on the premise that the organism’s changes in gene expression are largely adaptive, as in the glucose/lactose example. Our method is *unbiased*, in the sense that we do not select relevant genes *a priori*, but apply a uniform method to the interpretation of the expression of all genes represented in the model. This is useful because naïve interpretation of the expression of single genes can lead to incorrect predictions. For instance, the expression of genes for glucose transport does not imply the presence of glucose. *E. coli* growing solely in the presence of acetate will express glucose transporters, albeit at a lower level than it would if glucose were present.

To implement our approach we model the organism’s metabolic network using the method of flux balance analysis. Our key innovation is the way we use gene expression data to constrain reaction flux limits. Ideally, adaptive reductions in relative gene expression by the organism will be reflected in our model by reduced flux through key reactions. When we model growth we expect that the constraints placed by gene expression will not have a big impact when the corresponding *in silico* carbon source is used, but that model growth will be reduced for other *in silico* carbon sources. We therefore rank candidate nutrients by the extent to which their simulated growth is inhibited.

### Drawing Environmental Inferences from Gene Expression Data

Researchers may be motivated by biological, medical, engineering or ecological concerns to investigate an organism’s environment or its nutrient use. For example, understanding the *in vivo* environment of the pathogen *Mycobacterium tuberculosis* may provide clues toward the development of therapeutics, but it is difficult to study this environment directly. Analyzing the organism’s metabolic behavior may provide insight into its nutrient use and environment, but measurement of metabolic behavior *in vivo* is also difficult. Measurement of gene expression, on the other hand, is comparatively simple and, indeed, it is possible to measure the gene expression of *M. tuberculosis* during host infection [2]. The feasibility of using gene expression data to draw inferences about an organism’s environment has been previously demonstrated [3], [4], [5], [6]. In this paper, we develop a systematic method for relating measurements of gene expression to predictions of metabolic behavior.

What is novel in our approach is that the relevant biological knowledge is encapsulated in a metabolic model that links enzymatic reactions to genes, and that the inferences are based on the impact of gene expression on model growth. In some of the studies cited, prior biological knowledge was used to select genes of interest. In other studies, genes of interest were selected via clustering of expression profiles (or other computational methods) and then interpreted using a biological database such as Gene Ontology [7].

### Flux Balance Analysis (FBA)

We employ a flux balance approach to the modeling and analysis of the metabolism of *E. coli* [8]. (For a review of the scope of applications of FBA to *E. coli* see [9].**)** The central entity in the model is the reaction flux – the rate at which an enzymatic reaction proceeds. FBA models are valid on a time scale in which the reaction fluxes have reached steady state [10].

A core application of FBA models has been the accurate prediction of organism growth given nutrient uptake measurements. This approach, pioneered by Bernhard Palsson, makes the biological assumption that the organism is growing as rapidly as it can given its metabolic constraints**.** Computationally this corresponds to finding the maximum rate at which biomass – the *dry* constituents of a cell – can be produced subject to an FBA model’s constraints. These constraints include a minimum flux level for non-growth associated ATP maintenance to ensure viability of the *in silico* organism. This method and its underlying assumptions have proven successful for a range of organisms and growth conditions [8], [9], [10], [11], [12], [13].

### Regulation and the Modeling of Flux Limits

The first generation of FBA models made no attempt to model gene regulation. Non-uptake reactions typically had large non-limiting upper bounds on fluxes. Subsequently, regulatory FBA (rFBA) models [11], [12], [13] were developed. These models use a set of rules to explicitly model the role of isoenzymes, enzyme complexes, transcription factors and effector metabolites in controlling reaction flux. Evaluation of these rules with respect to metabolite and relative gene expression levels results in reaction upper flux limits being either non-limiting (on) or zero (off). Incorporation of gene expression data into metabolic models is an area of ongoing research [13], [14], [15].

Our method shares with that of C. Colijn e*t al.* [15] the use of gene expression data to set maximum flux limits. Allowing maximum flux limits to take on continuous values contrasts with approaches such as rFBA models which take a Boolean (on/off) approach to deriving flux limits from gene expression. Our approach can therefore model situations in which the down-regulation of a gene reduces, but does not abolish the flux through the corresponding reaction.

### Method Overview

Our method takes as input a *challenge* set of gene expression values obtained for growth on an unidentified *in vitro* nutrient and prioritizes a set of candidate nutrients in order of decreasing likelihood. In order to convert absolute expression values to relative ones the algorithm requires an estimate of the expression range for each gene. In our current implementation we use the maximum expression value for each gene over several gene expression sets for this estimate. We also require an FBA model which may include arbitrarily large flux limits.

**Figure 1:**
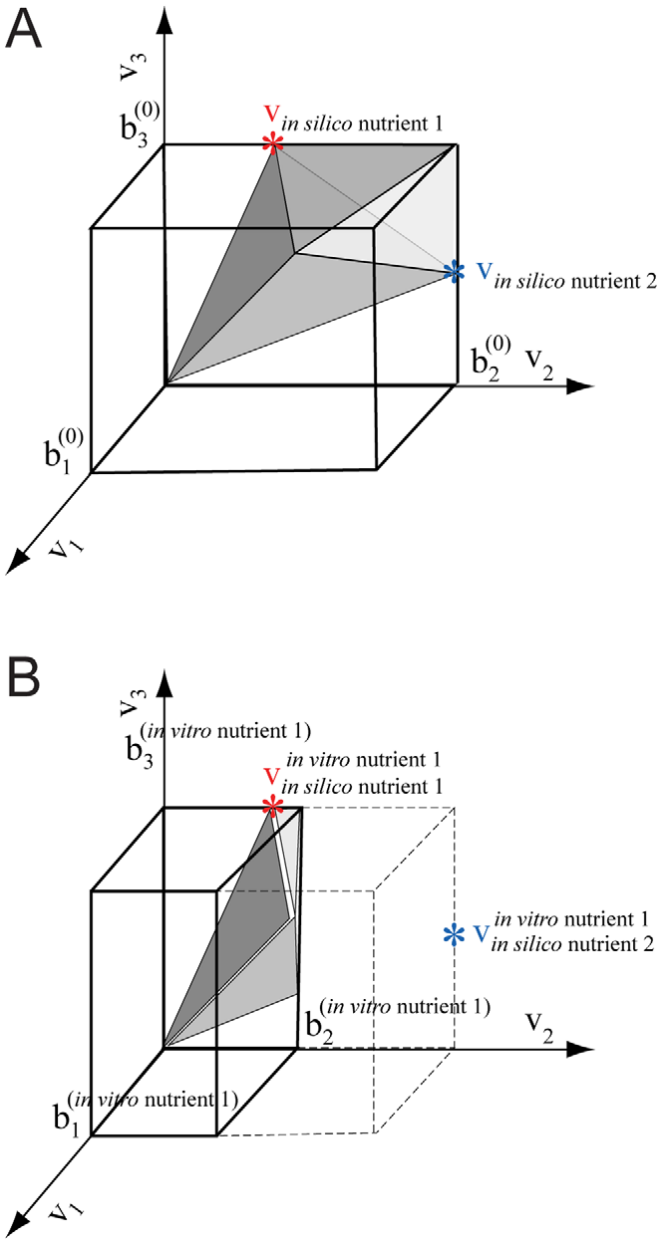
The principles of our method illustrated using a simple metabolic network. Flux limits in this figure are represented by a thin black outline. Reaction fluxes are represented as shaded regions with flux magnitude proportional to *thickness*. Flux direction is not indicated. **Panel A**. Creation of the baseline flux limits (corresponding to 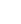 in Figure 2, Panel A)**.** Each reaction is given a flux limit corresponding to the maximum optimal flux solution over the two *in silico* nutrient uptake conditions. The shading is orange for *in silico* glucose growth, blue for *in silico* acetate and grey where the two solutions overlap. **Panel B.** Creation of the glucose expression-derived flux limits (corresponding to 
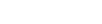
 in Figure 2, Panel B). Each flux limit shown in Panel A has been scaled by the level of gene expression for *in vivo* growth on glucose relative to the maximum gene expression for that reaction over both nutrient conditions. The arrows indicate two reactions for which gene expression was significantly lower on glucose than on acetate, resulting in significantly reduced flux limits. **Panel C**. Effect of the glucose expression-derived flux limits of Panel B on *in silico* glucose growth. The glucose optimal flux from Panel A (orange region) lies within the limits; biomass production is not changed. **Panel D.** Effect of the glucose expression-derived flux limits of Panel B on *in silico* acetate growth. The acetate optimal flux from Panel A (blue region) exceeds the flux limits for several reactions. (This is analogous to the optimal flux vector 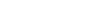 lying outside the flux cone in Figure 2, Panel B.) Hence the flux limits will lead to smaller optimal fluxes for these reactions and reduced biomass production. Relative biomass production is therefore smaller for *in silico* acetate than for *in silico* glucose, and we conclude that glucose is the more likely carbon source for the expression data.

**Figure 2:**
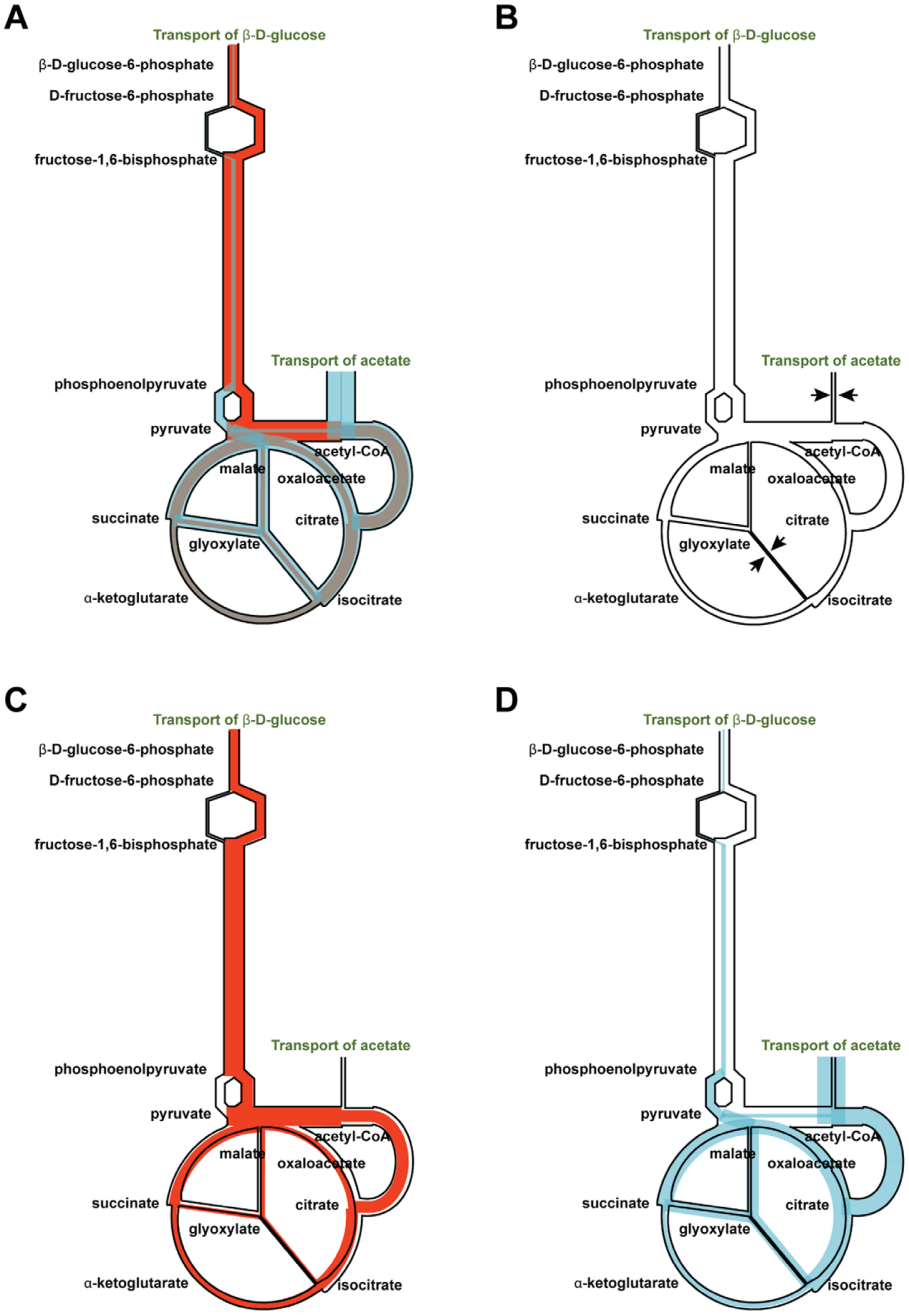
The principles of our method illustrated with flux cones. Only three of the many reaction fluxes are shown. For simplicity only two *in silico* candidate nutrients are represented. The figure does not correspond to actual experimental data. **Panel A.** Creation of the baseline flux limits, represented as a rectangular parallelpiped. Reaction fluxes must lie within the flux cone (grey area). Flux vectors producing maximal biomass for candidate nutrient *i* are indicated by colored asterisks and labeled 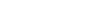 These solutions of the baseline FBA model constrained by *in silico* nutrient uptake lie on the surface of the flux cone. For each dimension *j* the baseline upper flux limit is denoted 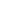 **Panel B.** Creation of the expression-derived flux limits by scaling the baseline flux limits. The upper flux limit for dimension *j* derived using expression data for the unknown *in vitro* nutrient *l* is denoted 
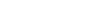
 and the solution vectors are denoted 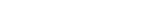 The baseline flux limits are indicated with dashed lines, the scaled limits are indicated by solid lines. In this hypothetical example the expression of the gene for the enzymatic reaction producing flux v_2_ is 40% of the maximal expression level for that gene under the other nutrient condition. The maximal flux for this reaction is set to 40% of its original level. This smaller flux cone represents the metabolic capabilities of the organism under the corresponding growth condition. The solution vector producing optimal biomass for nutrient 1 has not changed with the new flux limits, but the solution vector for nutrient 2 has been reduced in magnitude, with a consequent reduction in biomass production. Relative biomass production will be larger for nutrient 1 than for nutrient 2. We would therefore conclude that the *in vitro* nutrient *l* that gave rise to the expression profile is probably nutrient 1, rather than nutrient 2.

The key algorithmic steps in our method are as follows:

1. First, create a set of baseline flux limits for the metabolic model. Our procedure is intended to estimate the maximal flux capacity of each reaction for simulated growth over the selected set of in silico candidate nutrients (Figure 1, Panel A). This requires setting a realistic in vitro growth rate and computing the corresponding in silico nutrient supply rate.
2. Next, create expression-derived flux limits. These limits are specific to a challenge gene expression set, and are computed by scaling the baseline flux limits using the ratio of each gene’s expression level in the challenge condition to its maximum over several gene expression sets (see Figure 1, Panel B, and Figure 2, Panel B). The resulting flux limits reflect the organism’s adaptation to the unknown in vitro nutrient condition
3. Last, prioritize candidate nutrients for the challenge gene expression set. For each candidate nutrient:
  1. Find the expression-derived biomass production rate for the candidate nutrient by optimizing biomass production using the expression-derived flux limits and the baseline supply of that in silico nutrient.
  2. Compute the relative biomass production by dividing the expression-derived biomass production rate by the baseline biomass production rate (computed in step 1). The prioritization is accomplished by ordering the candidates by decreasing relative biomass production.

The ranking of the *in silico* nutrients provides the key measure of the success of our algorithm; if the correct *in silico* nutrient is ranked near the top only a few experiments will be necessary to validate it. We rank candidate nutrients by relative biomass production because we expect this to be high (near one) for the correct candidate nutrient, assuming that the organism’s expression pattern is well adapted to its nutrient environment. Relative biomass production will be reduced when there is a mismatch between the relative expression of a gene and the metabolic requirements for utilizing a candidate nutrient.

For example, *E. coli* requires the use of the glyoxylate shunt for growth on acetate, but not for growth on glucose. When grown on acetate it expresses the gene isocitrate lyase (*icl)* at a many-fold greater level than it does when grown on glucose. The upper flux limit for the corresponding glyoxylate shunt reaction will be near or at its maximum in the acetate expression challenge, but many-fold lower in the glucose expression challenge because of the difference in relative expression. Now consider the situation when growth on different *in silico* nutrients is evaluated using the glucose expression-derived flux limits. Under these conditions the flux capacity of the glyoxolate shunt reaction will be significantly reduced from the level needed for growth on acetate. Simulated growth on glucose will be essentially unimpaired, because growth on glucose does not require the glyoxolate shunt. However growth on acetate will be significantly reduced because of the now limiting rate of the glyoxolate shunt reaction. Even if the source of the expression data was unknown we could confidently state that glucose is a much stronger candidate for the carbon source than is acetate. This idea is illustrated in Figure 1, Panels C and D.

## Results

Our algorithm prioritizes candidate nutrients for their compatibility with an *in vitro* nutrient challenge gene expression set. The more compatible the *in silico* nutrient and the challenge expression data are, the larger the relative biomass production. We applied our method to a set of eighteen candidate carbon sources, using a set of six challenge gene expression data sets obtained from growth of *E. coli* on a subset of the candidate nutrients. The six challenge gene expression data sets were used to estimate maximum expression for each gene.

We present our results in two sections. The first section reports the rankings of the *in silico* nutrients for each challenge expression set and includes analyses that demonstrate the robustness and specificity of our algorithm. The second section compares simulated growth on an *in silico* nutrient across the six gene expression profiles, demonstrating the degree to which the gene expression profiles are tuned to their *in vitro* carbon sources.

### Rankings of the *in silico* Nutrients

**Figure 3:**
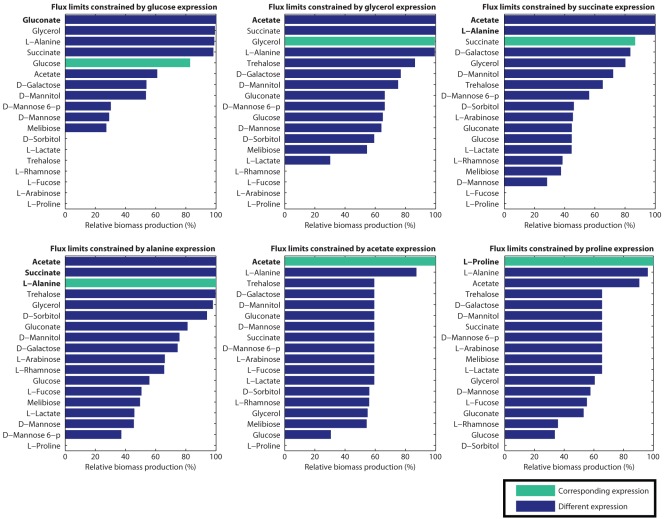
Priority ordering of candidate nutrients for each expression set. A panel for each of the six challenge expression sets presents the candidate nutrients ordered from top to bottom in order of decreasing relative biomass production. The length of the horizontal bars indicates the relative biomass production for the corresponding candidate nutrient for that challenge expression set. The bar for the *in silico* nutrient that corresponds to the *in vitro* carbon source for which expression was measured (the matching nutrient) is colored green. The other bars (for the non-matching nutrients) are blue. The order of candidate nutrients with equal relative biomass production is not meaningful. In particular this holds for the candidate nutrients with zero relative biomass production. Candidate nutrients with 100% relative biomass production are highlighted in bold.

Each panel in Figure 3 presents results for one of the six challenge expression sets. The candidate nutrients are sorted with the best candidate (with the largest percent relative biomass production) at the top. For cases where the *in silico* nutrient corresponds to the *in vitro* carbon source for the expression in the panel, the bar has a distinguishing green color. We refer below to such correspondences as *matching*, and to other pairs of candidate nutrient and challenge expression set as *non-matching*.

The rankings of the correct (matching) candidate nutrients are given in Table 1. The correct carbon source is one of the top three choices for five of the six challenge expression sets, and ranks fifth for one expression set. The mean ranking of the correct choice was 2.5 out of 18 (where we assigned an average ranking for ties) indicating a high degree of success.

**Table 1:** Ranking of the six challenge nutrients.

	orig. data	stochastic model		permutation model	
Challenge expression set	ranking	mean	std. dev.	mean	std. dev.
glucose	5	5.49	0.98	9.50	0.00
glycerol	3	3.74	1.87	9.50	0.00
succinate	3	3.78	1.23	9.50	0.00
Alanine	2	1.73	0.42	9.50	0.00
Acetate	1	1.20	1.26	9.50	0.00
proline	1	1.14	.77	9.50	0.00
**mean**	**2.50**	**2.85**		**9.50**	

Many approaches to analyzing gene expression limit their attention to genes that are strongly differentially expressed. To examine the effect of such filtering, we applied a cut-off on p-values calculated from a two-sample *t*-test comparing condition-specific gene expression with maximal expression for each reaction. Using a p-value cut-off at 0.05 to focus on significant expression changes caused a large reduction in the number of reactions for which scaling is applied. The resulting priority ordering of expression profiles and matching (see Figures S1 and S2) show that the biomass produced across the range of carbon sources increased with more carbon sources showing relative biomass production within 95% of the maximum production and a reduction in the ranking of the matching source in most cases.

The resulting loss of discriminatory power should not be surprising. Filtering of individual reactions in this manner fails to recognize the collective effect of a group of reactions on metabolic changes. Tools such as Gene Set Enrichment Analysis (GSEA) [16] attempt to achieve this by creating functional groupings. Our algorithm aims instead to use the relationship of the reactions within the structure of the metabolic network.

As can be seen from Figure 3, in many cases consecutively ranked nutrients are close in relative biomass production. We therefore tested whether the rankings would remain fairly stable despite the typical variations in gene expression found across biological or technical replicates. We undertook a stochastic analysis in which we simulated variation for all six expression data sets (see Methods). For each set of simulated expression data we ranked all the *in silico* nutrients, and then computed the means and standard deviations for each set of rankings. As can be seen from Table 1, the mean ranking over the six challenge sets increased slightly, going from 2.5 in the original analysis to 2.85 using the stochastic model.

Are the rankings of the matching *in silico* nutrients due to specific patterns of gene expression or to overall levels of gene expression? To answer this question we performed a simulation in which gene labels were randomly permuted (see Methods). The result (Table 1) was that the mean ranking of the correct nutrient over the six expression sets was 9.5 with a standard deviation of 0.0. With 18 choices, a random ordering of candidates would result in an average ranking for the correct nutrient of 9.5. This result clearly indicates that our original findings are due to the expression of specific genes, not overall gene expression.

This data should make it clear that our algorithm is able to significantly narrow the search for the carbon source corresponding to a set of gene expression data. Assaying candidate nutrients in prioritized order over the six expression sets would require an average of 2.5 tests with our algorithm. This is a four-fold improvement over the average 9.5 tests that would be required if candidate nutrients were assayed in a random order.

Our method is robust–adding perturbations to the expression values had no significant effect on the rankings. Furthermore, our method is sensitive to the expression of specific genes, since permutation of the genes resulted in a complete loss of discriminatory power.

### Optimality of Adjustment to Nutrient Conditions

**Figure 4:**
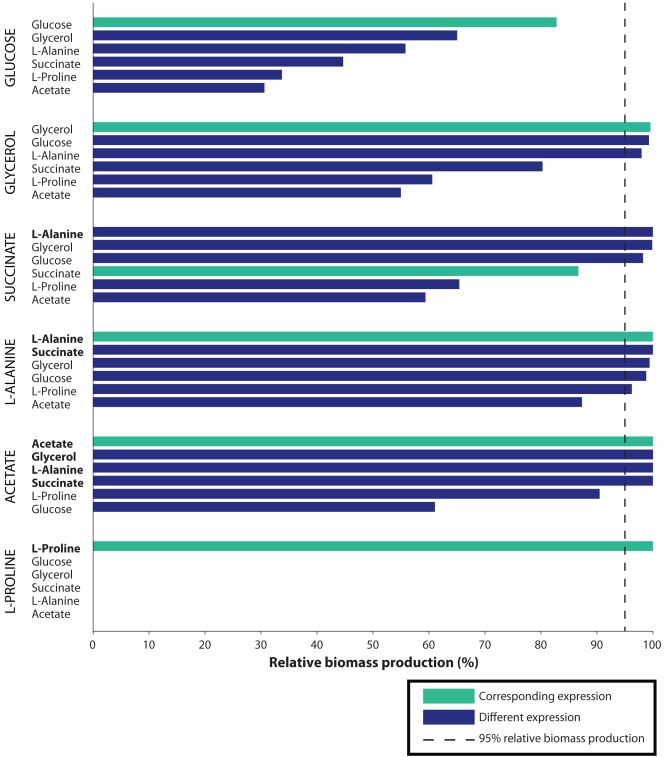
Near optimal growth is supported by most matching expression profiles. Each grouping of bars represents the relative biomass production of an *in silico* candidate nutrient with respect to the six challenge expression sets, which are in decreasing order from top to bottom. The vertical dotted line represents 95% relative biomass production**.** As in Figure 3, the length of the horizontal bars indicates the relative biomass production for the corresponding candidate nutrient for the indicated challenge expression set. The matching nutrients (green bars) reach the 95% threshold with higher frequency than the non-matching nutrients (blue bars), indicating the matching expression profiles are well adapted to that nutrient, while many of the non-matching profiles are not. The order of the candidate nutrients is that used in Table 1, and is not based on relative biomass production. Only *in silico* nutrients with a corresponding *in vitro* nutrient expression set are included in the figure. Candidate nutrients with 100% relative biomass production are highlighted in bold.

In this section we compare simulated growth of *E. coli* for *in silico* nutrients across the challenge gene expression sets, restricting ourselves to the challenge carbon sources. In Figure 4 for each *in silico* nutrient labeled vertically on the y-axis there is a group of bars representing the percent relative biomass production for that nutrient for each expression set. The expression sets are labeled horizontally on the y-axis and ordered by decreasing percent relative biomass production for each *in silico* nutrient.

Table 2 and Figure 4 show that *in silico* nutrients generally grow better when constrained by the matching expression set rather than one of the non-matching ones. The matching expression set was top ranked for five of six cases, and fourth for the remaining case. Averaging over all six candidate nutrients the mean for matching expression sets was 94.84%, dwarfing the 65.97% mean for non-matching expression.

**Table 2:** Relative biomass production for matching versus non-matching expression.

*in silico* nutrient	glucose	glycerol	succinate	alanine	acetate	proline	mean
matching expression	82.8	99.56	86.69	100	100	100	94.84
non-matching expression	45.97	78.61	84.59	96.35	90.31	0	65.97

The case where the matching expression set does not result in the greatest relative biomass production is succinate, and this case does rather poorly, with the relative biomass production of the matching expression set only slight greater than the mean for non-matching expression sets (86.69% as opposed to 84.59%). We suspect this may be due to the way in which succinate is metabolized, where it enters the TCA cycle directly, without any degradation reactions. The lack of degradation reactions reduces a key component of expression differentiation, which in turn reduces the specificity of our method.

The mean percent relative biomass production of 94.84% for matching expression sets shows that they do not impose much of a limitation on growth beyond that due to nutrient limitation, since by definition relative biomass production is the ratio of biomass production with both expression and nutrient constraints to biomass production with nutrient constraints alone.

The key results of this analysis are 1) gene expression for matching carbon sources supports nearly optimal growth and 2) growth constrained by non-matching gene expression is significantly reduced. Since the constraints due to matching gene expression have only a small effect on biomass production our method produces *in silico* growth close to that predicted by FBA models constrained by nutrient uptake alone. Such standard FBA models have been shown to accurately predict empirical growth rates of *E. coli* grown on several of the nutrients used as our sources of expression data [17]. The second result shows both that there is specificity to *E. coli’s* adjustment to each carbon source, and that our algorithm is sufficiently responsive to the corresponding changes in gene expression.

These results also show that for reactions critical to metabolic adjustment of varied carbon sources the baseline flux limits are neither dramatically too small nor too large, even though they were determined independently of the expression data. If the baseline flux limits were too small the expression-scaled limits would significantly impede optimal growth, and the relative biomass production could not approach one hundred percent. Similarly, if the baseline flux limits were too large, accurate discrimination between matching and non-matching expression would not be possible.

## Discussion

### Flux Limits

Setting meaningful flux limits for internal reactions – not just transport reactions – is critical for FBA modeling of situations in which reactions are enzyme-limited**.** Although traditional FBA techniques have been successfully used to model knockout organisms [11], [18], [19], they cannot model cases in which the increase or decrease of enzyme activity is relevant for predicting biomass production. Metabolic engineers have found cases in which decreasing enzyme activity increases the biosynthesis of a desirable product, but knocking out the corresponding gene results in an organism that is not viable [20].

An all-or-nothing approach to flux limits can result in incorrect predictions when gene expression data is applied to wild-type organisms [21]. Similarly Covert et al. [11] found that strict implementation of their statistical criteria for turning off reactions associated with down-regulated genes produced some incorrect predictions of loss of viability.

We believe that setting upper flux limits for internal reactions that take on values in a continuous range provides a realistic and powerful framework for applying gene expression data to metabolic models, and is a worthwhile alternative to using upper bounds that are either zero or non-limiting. Our core idea is that for conditions in which *in vivo* reaction fluxes are actually constrained by enzyme activity, the model’s reaction flux limits should also constrain *in silico* growth. Although reaction rates depend on the relative concentration of substrates and products, enzyme activity provides an upper limit on reaction rates. Actual reaction rates depend on the relative concentration of substrates and products, and may not attain this upper limit. As an early test of our ideas we used empirical activity values [22] to set flux limits and found we could discriminate between glucose and acetate as *E. coli* nutrient sources, using a simple model incorporating glycolosis, gluconeogenesis and the citric acid cycle.

The measurement of activity values is labor and time intensive, and a comprehensive set of such measurements is not available. Genome-scale gene expression measurement is, on the other hand, routine. We therefore developed a method that uses relative expression as a rough proxy for relative activity. Before discussing the logic and validity of our method we present our approach to deriving flux limits from expression values. First, we compute a set of baseline maximum flux rates (flux_max_) that conceptually play the role, for each reaction, of the maximal enzyme activity encountered over a set of experimental conditions. The condition-specific flux limits are obtained by scaling these values by relative expression. Mathematically we choose flux_max_ values for each reaction that are the maximum of any flux that might be encountered under optimal *in silico* growth conditions. We also determine minimal flux limits for all reversible reactions(details are in the Methods section). Notably, these limits generally lead to *in silico* model behavior that mimics the effect of rate-limited reactions.

We do not claim that every baseline flux limit approximates the actual maximum flux capacity of the corresponding reaction. The flux limits produced by our algorithm tend to underestimate the flux capacity of reactions that are always substrate-limited for the set of *in silico* candidate nutrients. This should not be problematic if the relative expression of corresponding genes is near one, which would be the case if their expression does not vary significantly over set of experiments used to determine the expression range. However for very small fluxes it may well be that there will always be sufficient enzyme, and that any reduction of these limits will have a deleterious effect. For example we needed to take special account of cofactors which, because they have very small coefficients in the biomass reaction, generally have small flux limits. Rather than removing cofactors from the biomass function one could set a relatively small minimum upper flux limit to simulate the presence of some enzyme. We found that using this approach with a minimum upper flux limit of.03 produced results essentially identical to those obtained with the alternative biomass function.

The results obtained by our method may be improved by setting flux limits using expression profiles obtained from cells adapted to specific nutrients over a number of generations. It has been observed that *E. coli* cells undergo adaptive evolution over several hundred generations to reach phenotypes predicted by FBA [17], and the expression changes that occur during this adaptive evolution should improve the performance of our method. Unfortunately, we could not find expression profile data sets of adaptively evolved *E. coli* on several nutrient sources (measured by the same lab to minimize spurious variation) which would be suitable for our method and which we could use to test this hypothesis.

### Assumptions and Approximations Underlying the Method

Enzyme activity is proportional to enzyme concentration. It can also be affected by changes in experimental conditions such as temperature and pH which alter the properties of the protein. Our method is appropriate for experiments designed to keep such variables nearly constant while allowing the nutrient source to vary. Such conditions justify our first approximation: the activity of an enzyme across experiments depends only on its concentration. We then make the further approximation that relative enzyme concentration is proportional to relative gene expression, while acknowledging that many factors affect enzyme concentration besides the level of the mRNA from which they are translated. Given these assumptions, the ratio between enzyme activity levels under two experimental conditions is a function of the relative expression of the corresponding genes.

The degree to which gene expression and enzyme activity are coupled is an empirical question. There is experimental evidence that a roughly linear relationship does hold for our system of interest. A study of nine *E. coli* central metabolism genes in both wildtype *E. coli* DF11 and a phosphoglucose isomerase (*pgi)* mutant found a relatively high Pearson correlation of.81 between the log ratio of transcripts and the log ratio of enzyme activities [22]. Another study [23] showed excellent correlation between log ratios for protein abundance and those for enzyme activities for *E. coli* grown on glucose, glycerol, gluconate and acetate. The one exception was isocitrate dehydrogenase (*idh*) which undergoes a four-fold reduction in activity due to phosphorylation when acetate is the carbon source. This is an example of knowledge that could be incorporated into our algorithm**.** The ability of our algorithm to achieve accurate results despite certain exceptions to our underlying assumptions should help make it clear that these assumptions do not need to hold absolutely for each gene, enzyme, reaction and carbon source.

On the other hand, application of our algorithm to eukaryotic systems in which posttranscriptional and posttranslational modifications play a significant role would require modification to incorporate additional data or biological knowledge. Attention must also be paid to other factors that can disrupt the relationship between gene expression and enzyme activity such as the intracellular localization of proteins and growth rate dependent differences in protein synthesis and degradation rates across conditions.

A final assumption made by our method, which is shared by other uses of FBA, is that the organism is optimizing the goal embodied in the model as an objective function. Our choice of goal, maximal growth (biomass production), is common for FBA models. Predictions based on biomass production as an objective function have been experimentally validated for the growth of *E. coli* and other organisms under a wide variety of environmental conditions [8], [9], [11], [23], [24]. Biomass production as an objective makes sense on evolutionary grounds for organisms competing in an environment in which optimal use of nutrients is an effective survival strategy**.** However under other circumstances organisms may adapt strategies for which growth is not a valid objective.

### Biological Conditions Necessary for Discrimination

The previous section discussed biological conditions that are necessary for the validity of our method. However, even if our method is valid additional conditions are necessary for its application to result in discrimination between nutrient sources. If all changes in flux result from changes in *mass action,* that is, from the relative concentration of substrates, products and modifiers our method will not avail, because flux limits do not play a limiting role. What our method needs for discrimination is regulation of some reactions by changes in enzyme concentration that result from changes in gene expression. With this type of regulation the reaction flux limits come into play for some nutrient conditions.

### Empirical Tests of Assumptions

As indicated previously, there is evidence in the literature that the biological assumptions underlying this project are valid to a sufficient degree for *E. coli* carbon metabolism. However in applying this method to different conditions it is important to test the validity of these assumptions empirically. In doing so it is helpful to keep in mind that most genes are not informative; specifically they do not undergo significant changes in relative expression. The authors of the paper from which we take our data considered that fewer than 300 out of approximately 4000 genes underwent significant change for the six experimental conditions [25]. For our method to be successful, significant increases in expression of metabolic genes must in most cases be markers of significant increases in enzyme activity, although the changes need not be strictly proportional. These changes in enzyme activity must also enable greater reaction flux for some genes.

One can directly determine the extent to which the relative mRNA expression is roughly proportional to both relative protein levels and to relative activity through concurrent measurements of gene expression, protein abundance and enzyme activity for key genes. RNA-Seq and quantitative RT-PCR for the measurement of gene expression for key genes may be desirable if gene chips produce results that are too noisy, having high levels of variance, and a more limited dynamic range. If relative enzyme protein abundance can be directly quantified, it can be used as input to our algorithm in place of mRNA expression.

The extent to which the regulation of key reactions in a pathway is due to changes in enzyme concentration versus changes in mass action can be tested using a method proposed by ter Kuile and Westerhoff [26].

### Conclusions

In this paper we presented a method to prioritize a set of candidate carbon sources with respect to their compatibility with a target gene expression profile**.** The method requires a metabolic network for the microorganism, and a small set of gene expression profiles measured under different nutrient conditions. We applied the method using eighteen candidate nutrients and a set of six *E. coli* expression profiles. We ranked the candidates using in turn each of the six profiles as the target. The correct nutrient was ranked in the top three for five profiles and was fifth for the other profile. Additional analyses showed these rankings to be robust to experimental variations in gene expression and to result from specific gene expression rather than general expression level. We also showed that the gene expression profiles are highly adaptive, with *in silico* organism growth being nearly optimal for matching *in silico* sources, while often being substantially reduced for non-matching sources. This result demonstrated that our baseline flux limits were well sized.

Our rankings depend on computing flux limits specific to experimental conditions. These flux limits are designed to approximate the constraints imposed on some reactions by enzyme activity. We analyzed the biological conditions that must hold for our approximation to be a valid one, and found they held for *E. coli* carbon metabolism.

One potential use of this method is the determination of nutrient use by pathogens in an intracellular environment. Direct determination of carbon nutrient sources may be significantly more challenging in such micro-environments than the measurement of gene expression. Another potential application of our method is its use as an aid to the creation of growth media for microorganisms. Development of such media is important for establishing laboratory growth conditions that can dramatically facilitate study of an organism, as illustrated by the development of the *candle method* for *P. falciparum* [27]. Since only a small percent of microorganisms identified in metagenomic studies are currently culturable in isolation [28] the application of *in vivo* expression data to help identify nutrients and environmental conditions (e.g., oxygen availability) necessary for their *in vitro* growth would benefit laboratory research.

## Methods

### Expression Data

We used gene expression data obtained by Liu et al. [25] and deposited in the Gene Expression Omnibus (GEO) database under accession number GSE2037. This data is comprised of the gene expression of *E. coli* grown separately on six different carbon sources – glucose, glycerol, succinate, L-alanine, acetate, and L-proline – as measured using Affymetrix GeneChip® *E. coli* antisense genome arrays. The data set consists of 15 samples: five samples of *E. coli* grown on glucose and two samples of *E. coli* grown on each of the five other carbon sources. Details of the experimental protocol followed and the normalization procedures applied to obtain the data can be found in [25]. We averaged the log-normalized expression of all samples in each condition and then exponentiated the averages to obtain the condition-specific expression used in subsequent analyses.

### Reaction Networks

The genome-scale model was created using the SBML file available with the supplementary material for Feist et al. [12]. We used the COBRA toolbox [29] to convert it to a COBRA model, and then transformed it into our internal format. Flux limits were changed as detailed in the following section.

In our calculations, we used a modified objective function that was created by removing the 15 metabolites specified as belonging to the category *Cofactors, Prosthetic groups and others* from the biomass reaction (see [12]). These metabolites are listed in Table 3, along with their original stoichiometry. Collectively they make up less than 2.9% of the biomass. We used this modified objective function because the reactions involved in synthesizing cofactors typically only require very small fluxes and, in our method, they have very small unscaled flux limits that cause bottlenecks during the scaling phase.

**Table 3:** Constituents removed from *i*AF1260 biomass.

Biomass Constituents	Amount (mm/gDW) in original biomass function
10-formlytetrahydrate	0.000223
2-octaprenyl-6-hydroxyphenol	0.000223
S-adenosylmethionine	0.000223
Coenzyme A	0.000576
FAD	0.000223
5,10-methylenetetrahydroflolate	0.000223
NAD	0.001831
NAD(P)	0.000447
protoheme	0.000223
pyridoxal 5′phosphate	0.000223
riboflavin	0.000223
siroheme	0.000223
tetrahydroflolate	0.000223
thiamine diphosphate	0.000223
undecaprenyl pyrophosphate	0.000055

### Baseline Flux Limits

We set baseline flux limits by the following procedure which is intended to estimate the largest range of fluxes encountered over the external conditions of interest, i.e., over growth on any of the 18 *in silico* nutrients we consider.

Following the standard FBA modeling framework (see, for example, [30]), we estimate the distribution of fluxes in *E. coli* in log-phase growth on a given nutrient by solving the following linear optimization problem:
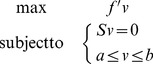
(1)where *v* is a length *n* vector representing the fluxes of the *n* reactions in the network, *f* is a length *n* vector representing the organism’s objective, *S* is a matrix containing the stoichiometries of the various reactions in the network (more precisely, 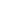 is the stoichiometric coefficient of metabolite *i* in reaction *j*), and *a* and *b* are vectors of lower and upper bounds on *v*, respectively. Let *j_biomass_* be the index of the reaction representing a unit of biomass in terms of its constituents. Then we set 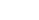: = 1, *f_i_* : = 0 for i ≠ *j_biomass_*, a typical objective for log-phase growth. By way of notation let F(*a, b*) be the optimal value 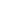 obtained with flux limit vectors *a* and *b*.

For a given nutrient *in silico k*, let *j_k_* be such that 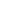 is the flux of the reaction that dictates the exchange of nutrient *k* with the external environment. We determine 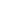 and 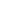 such that the biomass production corresponds to the experimentally measured growth rate of *E. coli* on nutrient *k*, as obtained from [25]. In the absence of experimentally measured growth rates for a particular nutrient, we assumed a nominal rate of 0.5 h^–1^. We denote this optimal biomass production rate as F*_k_*.

For each *in silico* nutrient *k*, with *a* and *b* set in this way, and the added constraint that biomass production must be at least 90% of its optimal (that is 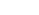: = .9F*_k_*), we successively maximize and minimize each flux subject to the constraints of the problem – a problem sometimes referred to as the max/min problem [31], [32], [33] – i.e., we solve
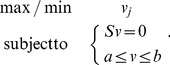
(2)


Let 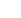 and 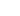 be the minimum and maximum reaction fluxes thus obtained, respectively. We set 
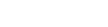
 and 
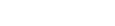
 and these vectors 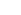 and 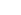 are the baseline flux limits. For a given *in silico* nutrient *k*, we take the baseline flux limits 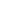 and 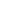 and appropriately set the component for exchange reaction *j_k_* as above. We denote the resulting lower and upper bound vectors 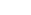 and 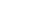 respectively.

### Determining Expression-derived Flux Limits

For each experimental condition *l* (with expression data set *l*), we determine flux limit vectors 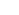 and 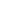
specific for that experimental condition. The calculation for a specific reaction depends on whether it is catalyzed by the product of a single gene, by more than one enzyme, or by a protein complex formed by several genes. If reaction *j* is catalyzed by an enzyme that is the product of a gene with expression 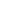 in experimental condition *l* we set 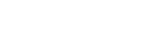 and 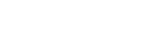. When reaction *j* is catalyzed by one of *n* enzymes (isozymes) formed by genes with expression 
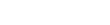
 we set 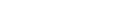 In the case that reaction *j* is catalyzed by a protein complex formed by genes with expression 
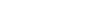
 we set 
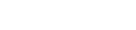
 For nested relationships, we apply these rules repeatedly. We denote the lower and upper bound vectors corresponding to *in silico* nutrient *k* and experimental condition *l,* by 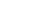 and 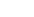 respectively, and set the component of the exchange reaction *j_k_* as above.

### Computing Relative Biomass Production

Relative biomass production for an expression set *l* and an *in silico* nutrient *k* is denoted by RBP(*l,k)* and is defined as
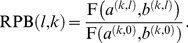
(3)


For convenience the results are reported as percents.

### Stochastic Analysis

For each iteration of the stochastic analysis, normally distributed random values with mean zero were added to the log base two of the expression values. The expression values were then transformed back via exponentiation. The relative biomass was recomputed for each combination of expression set and *in silico* nutrient. The candidate nutrients were ordered for each challenge expression set. This procedure was repeated 1000 times**.** Given a gene *g* and a set of *n* replicate expression measurements 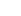
(base 2) for expression set *l*, the standard deviation of the Gaussian distribution was 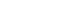 Division by 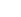 is a computational shortcut for averaging *n* random values from a Gaussian distribution with standard deviation 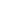 justified by a well-known result on the sum of independent normally distributed random variables**.**

### Permutation Analysis

For each iteration of the permutation analysis, the set of expression values for all 3797 *E. coli* genes in the data set was permuted and the relative biomass was recomputed for each combination of expression set and *in silico* nutrient. The candidate nutrients were ordered for each challenge expression set. This procedure was repeated 1000 times.

We implemented our method using Gurobi Optimizer (Gurobi Optimization, Houston, TX) and MATLAB (The MathWorks, Inc., Natick, MA).

## Supporting Information

Figure S1 **Priority ordering of candidate nutrients for each expression set with p-value filtering.** This figure is the analog of Figure 3 for the case where p-value filtering with a cut-off of 0.05 has been applied to the expression sets.
(EPS)

Figure S2 **Relative biomass production for each expression set with p-value filtering.** This figure is the analog of Figure 4 for the case where p-value filtering with a cut-off of 0.05 has been applied to the expression sets.
(EPS)
