# Correction: Inferring Carbon Sources from Gene Expression Profiles Using Metabolic Flux Models

**DOI:** 10.1371/annotation/139857f3-5a05-4a23-9bfe-a77aafbce54d

**Published:** 2012-08-24

**Authors:** Aaron Brandes, Desmond S. Lun, Kuhn Ip, Jeremy Zucker, Caroline Colijn, Brian Weiner, James E. Galagan

There was an error in the placement of Figures 1 and 2. The figures were inadvertently swapped. The figure legends are in the correct locations. 

